# Angle-Independent Color Changes in Elastomer-Immobilized Non-Close-Packed Colloidal Amorphous Films Under Stretching

**DOI:** 10.3390/polym18030382

**Published:** 2026-01-31

**Authors:** Yuna Hirano, Koyuki Hayashi, Toshimitsu Kanai

**Affiliations:** 1 Graduate School of Engineering Science, Yokohama National University, 79-5 Tokiwadai, Hodogaya-ku, Yokohama 240-8501, Kanagawa, Japan; yuna.hirano.0405@gmail.com; 2 College of Engineering Science, Yokohama National University, 79-5 Tokiwadai, Hodogaya-ku, Yokohama 240-8501, Kanagawa, Japan; hayashi-koyuki-xr@ynu.jp

**Keywords:** structural color, colloidal amorphous structure, elastomers, stimuli-sensitive materials, color tuning

## Abstract

Colloidal amorphous structures comprise short-range ordered arrays of monodisperse submicrometer-sized particles. They exhibit angle-independent structural color and hence are expected to be promising candidates for advanced color materials. In particular, non-close-packed colloidal amorphous structures embedded in soft polymers can alter the angle-independent color through stimuli-induced volume changes in the polymer. Consequently, such materials should have significant potential for application in sensor devices. This paper reports the preparation of an elastomer-immobilized non-close-packed colloidal amorphous film with an angle-independent color using a hydrogel-immobilized non-close-packed colloidal amorphous film as the starting material. The swelling solvent (i.e., water) in the hydrogel film was replaced with a hydrophilic elastomer precursor solution, which was photopolymerized to immobilize the colloidal amorphous structure with the separated particles within the elastomer film. The color of the elastomer-immobilized non-close-packed colloidal amorphous film was angle-independent and was easily altered under stretching. Furthermore, hydrophilic carbon black dispersed well in the hydrophilic elastomer precursor solution, improving the saturation of the resultant elastomer-immobilized non-close-packed colloidal amorphous film. The flexible nature of the prepared film should allow it to be attached to curved surfaces, thereby promoting its application as a simple strain sensor to express invisible strains through color changes.

## 1. Introduction

Color arising from the scattering or reflection of light by ordered nanostructures is known as structural color [1,2,3] and is often observed in nature, such as in the wings of morpho butterflies and jewel beetles, and in the skin of chameleons [4,5,6,7,8]. Unlike conventional pigments and dyes, structural colors possess distinct features, including durability against ultraviolet (UV) light and color tunability in response to structural changes, rendering them highly promising for use in advanced color materials and sensors [9,10,11,12,13]. Colloidal crystals, consisting of periodic arrays of monodispersed submicrometer-sized particles characterized by long-range order and brilliant reflective colors owing to Bragg reflections, are among the most widely studied artificial structural colors [14,15,16,17]. In particular, non-close-packed colloidal crystals embedded in soft polymers exhibit significant color tunability owing to the changes in particle spacing that result from stimuli-induced alterations in the polymer volume. Examples of such external stimuli include changes in temperature [18,19], solvent [20,21], and mechanical stress [22,23]. These materials are therefore expected to be applicable in colorimetric sensors that detect changes in environmental conditions. However, according to Bragg’s law [14,15], the color of colloidal crystals varies with the viewing angle, which may limit their application.

Colloidal amorphous structures, which comprise short-range ordered arrays of monodispersed colloids, are artificial structural materials that exhibit angle-independent colors [24,25,26]. Generally, colloidal amorphous arrays comprise monodisperse particles that are in contact with one another in the dry state. Although their color is not as vivid as that of colloidal crystals, saturation can be improved by adding black substances, such as carbon black [27,28], Fe_3_O_4_ [29], and polydopamine [30] to suppress incoherent scattering. Recently, our group discovered a non-close-packed colloidal amorphous structure with an angle-independent color between the crystalline and random phases in a charged colloidal suspension [31]. The addition of a small amount of salt to these charge-stabilized colloidal crystals (i.e., low-density crystalline arrangements of charged colloids in water) induced a transformation into an amorphous structure with an angle-independent color, owing to the reduced electrostatic repulsion between particles. Furthermore, the non-close-packed colloidal amorphous structure was immobilized in a stimuli-responsive hydrogel film while maintaining the separated particle structure. This allowed for a notable change in the angle-independent structural color according to environmental conditions, such as variations in the swelling solvent [31] and temperature [32].

In this study, an elastomer-immobilized non-close-packed colloidal amorphous film exhibiting angle-independent color is prepared, for the first time, using a hydrogel-immobilized non-close-packed colloidal amorphous film as the starting material. The water-swelling solvent in the hydrogel film is replaced with a hydrophilic elastomer precursor solution, which is subsequently photopolymerized to immobilize a colloidal amorphous structure with the separated particles within the elastomer film. The angle-independent behavior of the elastomer film color is evaluated, and the effect of stretching is investigated. Furthermore, the dispersion of hydrophilic carbon black in the hydrophilic elastomer precursor solution is performed to improve the saturation of the resulting elastomer-immobilized non-close-packed colloidal amorphous film.

## 2. Materials and Methods

### 2.1. Preparation of the Hydrogel-Immobilized Non-Close-Packed Colloidal Amorphous Film

**Figure 1:**
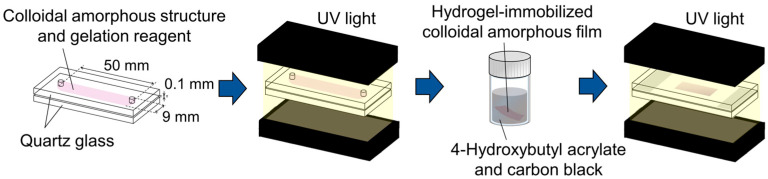
Schematic outlining the preparation of the elastomer-immobilized non-close-packed colloidal amorphous film.

A hydrogel-immobilized non-close-packed colloidal amorphous film was prepared according to a previously reported method [31,32]. Specifically, an aqueous suspension of monodisperse polystyrene particles with a diameter of 160 nm (5016 B, Thermo Fisher Scientific, Waltham, MA, USA) and 10 wt.% ion exchange resin (AG501-X8(D), Bio-Rad, Hercules, CA, USA) was gently stirred for 2 weeks to promote deionization. The resulting charge-stabilized colloidal crystals were then subjected to centrifugation to increase the particle concentration. The gelation reagent was prepared by dissolving N-methylolacrylamide (NMAM, FUJIFILM Wako Pure Chemical Corp., Tokyo, Japan) and N-isopropylacrylamide (NIPAM, FUJIFILM Wako Pure Chemical Corp., Tokyo, Japan) monomers, along with the N,N’-methylenebisacrylamide (BIS, FUJIFILM Wako Pure Chemical Corp., Tokyo, Japan) cross-linker, and the 2,2′-azobis[2-methyl-N-(2-hydroxyethyl) propionamide] (VA, FUJIFILM Wako Pure Chemical Corp., Tokyo, Japan) photoinitiator in ultrapure water (Merck KGaA, Milli-Q system, Darmstadt, Germany). The charge-stabilized colloidal crystals, gelation reagent, and ultrapure water were then mixed in a square cuvette (1 cm length, 1 cm width, 4.5 cm height) to give a total volume of 0.5 mL, along with polystyrene particle, NMAM, NIPAM, BIS, and VA concentrations of 9.63 vol.%, 400 mM, 400 mM, 40 mM, and 0.35 mM, respectively. An aqueous solution of NaCl (10 mM) was then added to the cuvette in 0.5 µL aliquots until the iridescence of the colloidal crystals disappeared. The aqueous, suspended colloidal amorphous structures (NaCl concentration = 110 µM) and colloidal crystals (NaCl concentration = 0 µM) were bubbled with argon gas for 5 min and subsequently sealed in a flat capillary cell (9 mm channel width, 50 mm length, 0.1 mm height) composed of quartz glass (Figure 1). The top and bottom faces of the cells were irradiated with UV light (MBRL-CUV7530, MORITEX SCHOTT, Saitama, Japan) for 1 h to promote photopolymerization of the dissolved gelation reagent. The resulting hydrogel films containing non-close-packed colloidal amorphous structures and colloidal crystals were subsequently removed from the cells.

### 2.2. Preparation of the Elastomer-Immobilized Non-Close-Packed Colloidal Amorphous Film

The hydrogel-immobilized non-close-packed colloidal amorphous and colloidal crystal films were cut into rectangular specimens measuring 20 mm × 8 mm (length × width). The hydrogel-immobilized non-close-packed colloidal amorphous film was immersed in a hydrophilic elastomer precursor, namely 4-hydroxybutyl acrylate (4HBA, Tokyo Chemical Industry, Tokyo, Japan) containing 1 wt.% hydrophilic carbon black (Aqua-Black^®^162, Tokai Carbon Co., Ltd., Tokyo, Japan), for 3 min. The film was then placed in a screw-tube bottle containing 4HBA and 1 vol.% photoinitiator (DAROCUR 1173, BASF Japan Ltd., Tokyo, Japan) and mixed using a vortex mixer (G560, Scientific Industries, Inc., Bohemia, NY, USA) for 5 min to remove any excess carbon black from the film surface. The hydrogel-immobilized non-close-packed colloidal amorphous films, with and without carbon black, and the hydrogel-immobilized non-close-packed colloidal crystal film were transferred to screw-tube bottles containing 4HBA and 1 vol.% photoinitiator (DAROCUR 1173), then allowed to stand for 1 d. Glass slides were coated with silicone oil (KF-96A-50CS, Shin-Etsu Chemical Co., Tokyo, Japan) as a release agent. The hydrogel films swollen in the 4HBA solution were sandwiched between two glass slides using 0.15 mm-thick silicone rubber sheets as spacers. UV light (MBRL-CUV7530) was used to irradiate both the top and bottom of the film for 3 min to prepare elastomer-immobilized non-close-packed colloidal amorphous films with and without carbon black, along with the corresponding elastomer-immobilized non-close-packed colloidal crystal film.

### 2.3. Characterization

The reflection and transmission spectra were measured using a fiber spectrometer (Fastevert S-2630, Soma Optics, Tokyo, Japan) and a UV-visible spectrophotometer (V-670, Jasco Corp., Tokyo, Japan), respectively. The elastomer-immobilized non-close-packed colloidal amorphous and colloidal crystal films were pinched at both edges and stretched. Photographic images of the films at different degrees of stretching were captured at various viewing angles under illumination with a light-emitting diode (LED, MR-2, AS ONE, Osaka, Japan). These images were recorded using a digital camera (IXY DIGITAL 800 IS, Canon, Tokyo, Japan).

## 3. Results and Discussion

**Figure 2:**
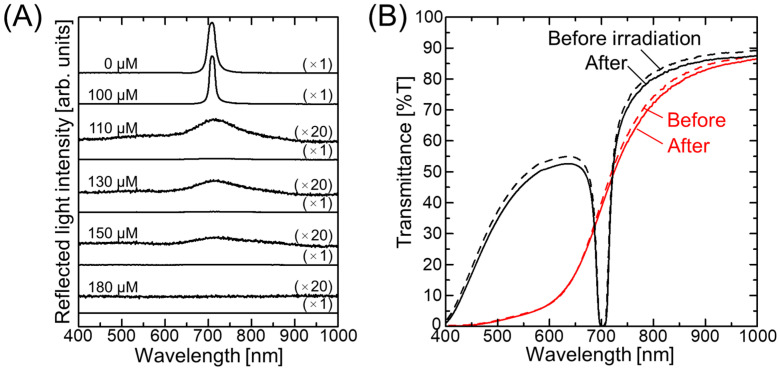
(**A**) Changes in the reflection spectrum at normal incidence for the charged colloidal suspension with increasing salt concentration. (**B**) Transmission spectra recorded at normal incidence for the non-close-packed colloidal crystal with a salt concentration of 0 µM (black line) and for the amorphous structure with a salt concentration of 110 µM (red line) before and after UV light irradiation.

Figure 2A shows the change in the reflection spectrum at normal incidence for the charged colloidal suspension with increasing salt concentration. The suspension prepared in the absence of salt, i.e., containing the charge-stabilized colloidal crystals, exhibited a pronounced Bragg reflection at ~710 nm, which was attributed to the (111) lattice planes of the face-centered cubic (FCC) structure [33]. Upon increasing the salt concentration to 110 µM, the reflection peak was significantly diminished, and a broad peak originating from the amorphous structure was observed at 20× magnification (see Figure 2A). The intensity of this peak was further reduced at higher salt concentrations and was not observable at concentrations >180 µM. Subsequently, these aqueous, suspended non-close-packed colloidal crystals and amorphous structures were immobilized in hydrogel networks through photopolymerization of the dissolved gelation reagent. Owing to the Bragg reflection from the FCC (111) lattice planes, the non-close-packed colloidal crystal (NaOH concentration = 0 µM) showed a deep dip in its transmission spectrum prior to UV light irradiation, and the spectral profile was almost preserved after irradiation, with a <5% decrease in transmittance (Figure 2B). In contrast, the non-close-packed colloidal amorphous structure (NaCl concentration = 110 µM) exhibited a characteristic transmission spectrum of an amorphous structure, wherein the low transmittance observed in the short-wavelength range increased sharply close to the Bragg wavelength [31]. Following UV light irradiation, the spectral profile was largely maintained, with a <3% decrease in transmittance being observed. These results confirm successful preparation of the hydrogel-immobilized non-close-packed colloidal crystal and amorphous films, consistent with previous reports [31,32].

**Figure 3:**
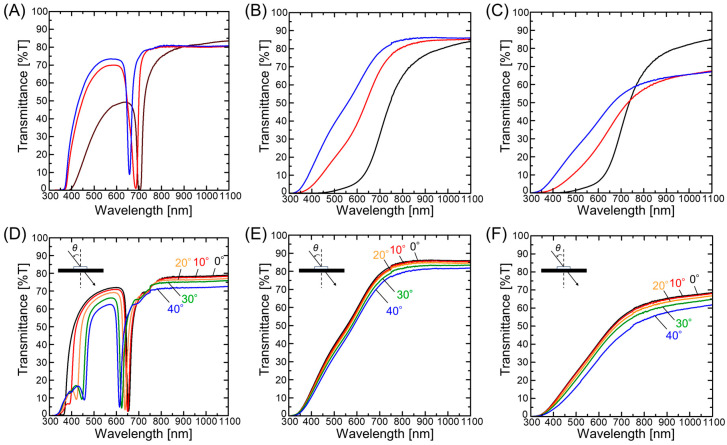
Transmission spectra recorded at normal incidence for (**A**) the hydrogel-immobilized non-close-packed colloidal crystal film and the hydrogel-immobilized non-close-packed colloidal amorphous films (**B**) without and (**C**) with incorporated carbon black (black line: prior to solvent replacement; red line: after solvent replacement; blue line: after photopolymerization). Transmission spectra recorded for (**D**) the elastomer-immobilized non-close-packed colloidal crystal film and the elastomer-immobilized non-close-packed colloidal amorphous films (**E**) without and (**F**) with incorporated carbon black at different incident light angles.

The water in the hydrogel-immobilized non-close-packed colloidal amorphous film was then replaced with a hydrophilic elastomer precursor solution (4HBA), and subsequent photopolymerization was performed to yield an elastomer-immobilized non-close-packed colloidal amorphous film. For comparison, Figure 3A shows the transmission spectra recorded for the hydrogel-immobilized non-close-packed colloidal crystal film at normal incidence before and after solvent replacement with 4HBA and subsequent photopolymerization. Following solvent replacement with 4HBA, the transmission spectrum exhibited a blue shift, primarily due to the reduced particle spacing in the shrunken hydrogel. Additionally, the spectral dip became shallower, indicating a slight deterioration in the crystalline structure. Upon UV light irradiation of the 4HBA absorbed by the hydrogel film, a polymer network developed, solidifying and immobilizing the gel structure. As a result, an elastomer film that captured the colloidal crystals while maintaining particle separation was obtained. Following UV light irradiation, the spectral dip underwent a further blue shift and became shallower, mainly due to the reduction in particle spacing and deterioration of the crystal structure, respectively. Similarly, the hydrogel-immobilized non-close-packed colloidal amorphous film exhibited a blue shift in its spectrum after solvent replacement and subsequent photopolymerization (Figure 3B), indicating a decrease in particle spacing. The transmittance gradient in the spectrum decreased after these processes, suggesting a slight deterioration in the amorphous structure. Upon the addition of hydrophilic carbon black to 4HBA, the transmittance decreased over the entire wavelength range (see Figure 3C), owing to the absorption of incoherent light by carbon black. Moreover, the elastomer-immobilized non-close-packed colloidal crystal film exhibited an angle-dependent spectral change, wherein the dip blue-shifted with an increase in the incident light angle (*θ*), consistent with Bragg’s law (Figure 3D). In contrast, the elastomer-immobilized non-close-packed colloidal amorphous films prepared both with and without carbon black did not exhibit such spectral shifts, as shown in Figure 3F and Figure 3E, respectively.

**Figure 4:**
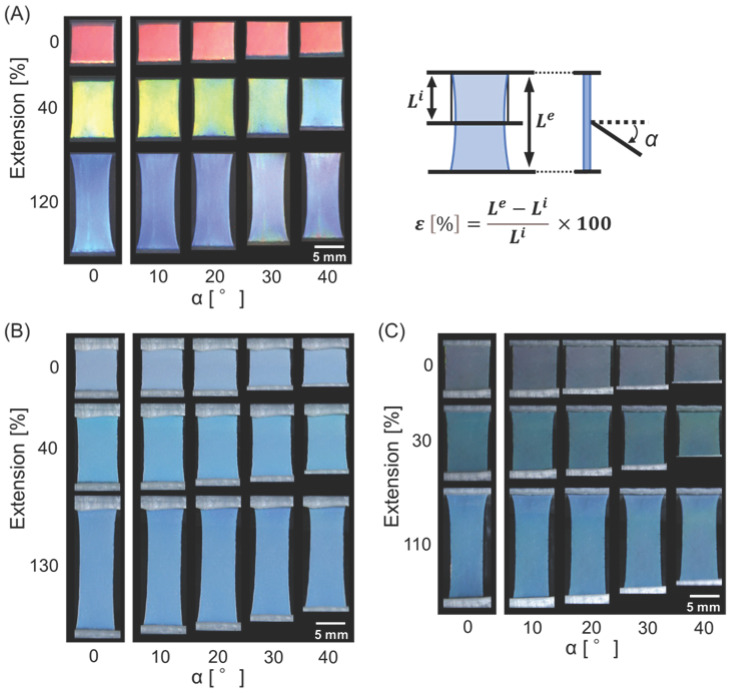
Color changes in (**A**) the elastomer-immobilized non-close-packed colloidal crystal film and the elastomer-immobilized non-close-packed colloidal amorphous films (**B**) without and (**C**) with incorporated carbon black under stretching and observed at different viewing angles.

The highly stretchable nature of the elastomer allows the spacing between particles in the film to change, thereby enabling the elastomer color to be easily tuned upon stretching. As shown in Figure 4A, the color of the elastomer-immobilized non-close-packed colloidal crystal film, which was red in the initial state, changed to yellow and blue under stretching degrees (*ε* = ((*L*^e^ − *L*^i^)/*L*^i^) × 100, *L*^e^ = film length at extension, *L*^i^ = film length without extension) of 40% and 120%, respectively. However, the viewing angle (α (°) = angle in the longitudinal direction of the film relative to the normal of the film surface) had a significant effect on the observed color under each stretching condition. In contrast, the elastomer-immobilized non-close-packed colloidal amorphous film exhibited an angle-independent color change upon stretching, although it was not as vivid as that of the colloidal crystal system (Figure 4B,C). However, the saturation was improved by the addition of carbon black, as shown in Figure 4C, and the film displayed green and blue colors at stretching degrees of 30% and 110%, respectively, without angle dependence. Upon release from stretching, the film returned to its original size and color. The film was fully reversible, and the color change was repeatable.

## 4. Conclusions

An elastomer-immobilized non-close-packed colloidal amorphous film with angle-independent color changes was successfully prepared using a hydrogel-immobilized non-close-packed colloidal amorphous film as the starting material. The water in the hydrogel film was easily replaced with a hydrophilic elastomer precursor solution, 4HBA, while maintaining the amorphous structure. Subsequent photopolymerization enabled the immobilization of the colloidal amorphous structure with the separated particles within the elastomer film. Unlike the colloidal crystal system, the elastomer-immobilized non-close-packed colloidal amorphous film did not exhibit a spectral shift upon changing the incident light angle, resulting in an angle-independent color. Furthermore, the dispersion of hydrophilic carbon black in 4HBA produced an elastomer-immobilized non-close-packed colloidal amorphous film containing carbon black, which exhibited improved saturation. Due to the highly stretchable nature of this structure, the film color was easily altered upon stretching. Specifically, the color changed to green and blue under stretching degrees of 30% and 110%, respectively, without any angle dependence being observed. However, the direct observation of the non-close-packed particle arrangement immobilized in the soft polymer remains a challenge for future studies. The present results suggest that the prepared film could be attached to curved surfaces and applied as a simple strain sensor to express invisible strains through color changes. Moreover, when fabricated over a large area, this film has the potential to visualize the spatial distribution of strain.

## Data Availability

The original contributions presented in this study are included in the article. Further inquiries can be directed to the corresponding author.

## Abbreviations

The following abbreviations are used in this manuscript:

UV	ultraviolet
NMAM	N-methylolacrylamide
NIPAM	N-isopropylacrylamide
BIS	N,N’-methylenebisacrylamide
VA	2,2′-azobis[2-methyl-N-(2-hydroxyethyl) propionamide]
4HBA	4-hydroxybutyl acrylate
LED	light-emitting diode
FCC	face-centered cubic
